# Novel alphavirus-based vaccine induces strong immune responses against prostate-specific antigen (PSA) and mediates tumor cell elimination in a mouse model of prostate cancer

**DOI:** 10.1186/2051-1426-1-S1-P221

**Published:** 2013-11-07

**Authors:** Vladimir Ryabov, Peter Pushko, Irina Tretyakova, Rikka Saito, Richard B Alexander, Elena N Klyushnenkova

**Affiliations:** 1 Surgery, University of Maryland, Baltimore, MD, USA; 2 Medigen Inc., Frederick, MD, USA

## 

Prostate cancer is the second most frequently diagnosed cancer in men worldwide and sixth leading cancer-related cause of death in males. Treatments for recurrent prostate cancer are only rarely curative, and the majority of patients eventually demonstrate progressive disease. Animal and clinical studies suggested that immunological approaches can be useful for prostate cancer treatment and prevention. The development of immunotherapy for prostate cancer based on the induction of autoimmunity to prostate-specific differentiation antigens is an attractive concept. Prostate specific antigen (PSA) is a serine protease of the glandular kallikrein gene family with highly restricted expression on prostate tumor cells and normal prostate. Previous attempts to develop PSA-based vaccines for prostate cancer primarily using poxvirus and adenovirus vectors resulted in promising clinical and immune responses. However, these approaches had limitations due to pre-existing antibody responses to viral vectors and insufficient safety. Previously we have constructed novel non-replicative alphavirus vector platform derived from live attenuated human TC-83 IND vaccine against Venezuelan equine encephalitis (VEE) virus infection. In earlier studies, vaccines based on alphaviruses demonstrated efficiency in targeted gene delivery to DC and induction of protective CTL immunity against several human viral pathogens and breast cancer in a rodent animal model. In this study we tested novel TC-83 based PSA-encoding alphavirus vaccine (vVLP-PSA) against prostate cancer using transgenic mouse model with established neonatal tolerance to human PSA. Preventive vaccination using vVLP-PSA demonstrated induction of strong PSA-specific CD8+ T-cell response as detected by IFNγ ELISPOT assay and intracellular cytokine staining. Potent expansion of PSA-specific CD8+ effector T-cells was found in peripheral blood and spleens of vaccinated mice using PSA peptide-loaded MHC I dextramers. vVLP-PSA vaccination also induced PSA-specific antibody production in immunized mice. Injection of TRAMP tumor cells expressing PSA into vaccinated mice resulted in elevated infiltration of tumor site with CD8+ T-cells and rapid elimination of PSA-expressing tumor cells during first several weeks after tumor challenge. Tumor survival analysis revealed tendency for delayed tumor growth in vVLP-PSA immunized mice. Altogether, our data demonstrate that TC83-based vaccine encoding PSA induces strong PSA-specific CTL response and mediates efficient elimination of antigen-expressing prostate tumor cells promptly after tumor challenge.

